# Alcohol consumption among university students in Ethiopia: a systematic review and meta-analysis of epidemiological studies

**DOI:** 10.3389/fpubh.2025.1513242

**Published:** 2025-09-09

**Authors:** Sisay Abebe Debela, Yonatal Mesfin Tefera, Endashaw Abebe Debela, Mesfin Gebrehiwot, Anmut Endalkachew Bezie, Ejeta Batu Dadi, Chala Daba

**Affiliations:** ^1^Department of Public Health, College of Medicine and Health Sciences, Salale University, Fiche, Ethiopia; ^2^Adelaide Exposure Science and Health, School of Public Health, University of Adelaide, Adelaide, SA, Australia; ^3^School of Internal Medicine, Adama Hospital Medical College, Adama, Ethiopia; ^4^Environmental Pollution Monitoring and Study Desk, Ethiopian Environmental Protection Authority, Addis Ababa, Ethiopia; ^5^Department of Occupational Health and Safety, College of Medicine and Health Sciences, Wollo University, Dessie, Ethiopia; ^6^Oromia Health Bureau, Addis Ababa, Ethiopia; ^7^Department of Environmental Health, College of Medicine and Health Sciences, Wollo University, Dessie, Ethiopia

**Keywords:** alcohol consumption, university students, Ethiopia, systematic review and meta-analysis, students

## Abstract

**Background:**

Alcohol consumption among University students in Ethiopia is a pressing public health concern. Many existing studies tend to focus on students across all educational levels, including combinations of high school, college, and University students, and determining substance use behaviors, such as Kchat chewing, cigarette smoking, and alcohol consumption. This approach lacks a comprehensive national perspective, particularly with regard to University students. Notably, there is no research available that assesses the extent of alcohol consumption among university students in Ethiopia on a national level. This systematic review and meta-analysis aim to provide comprehensive insights into the prevalence of alcohol use among University students and the factors influencing their drinking behaviors.

**Methods:**

We conducted a comprehensive electronic search across various international databases and libraries, including PubMed/MEDLINE, Hinari, Science direct, African Journals Online and Google Scholar, to identify relevant published reports. The study strictly adhered to the Preferred Reporting Items for Systematic Reviews and Meta-Analysis Protocols (PRISMA) guidelines. Analysis was performed using STATA 14 software, employing a random effects model to estimate the pooled effect with a 95% confidence interval (CI). Heterogeneity was visually assessed using forest plots. Additionally, publication bias was evaluated through funnel plots and Egger’s and Begg’s tests.

**Results:**

We conducted an analysis of 15 studies involving a total of 10,500 University students in Ethiopia to determine the prevalence of alcohol consumption. Sample sizes across these studies ranged from 188 to 1,239 participants. The pooled prevalence of alcohol consumption was 36.5%. Students who had friend’s currently consuming alcohol were found to be twice as likely to engage in alcohol consumption. Additionally, students in Universities who smoke cigarette were also twice as likely to consume alcohol compared to non-smokers. Furthermore, students with family members who drink alcohol were more likely to consume alcohol compared to those from families without this behavior. The study identified *Kchat* chewing habit as an additional factor influencing alcohol consumption among University students in Ethiopia.

**Conclusion:**

Addressing alcohol consumption in these students requires comprehensive interventions that consider these diverse factors to promote responsible drinking and student well-being.

## Introduction

Alcohol consumption among University students is a global concern due to its potential health, social, and academic implications (1). The transition from adolescence to adulthood is marked by the allure of newfound freedom and responsibilities, making this group particularly vulnerable to alcohol-related issues. Worldwide, alcohol consumption among University students follows a diverse trend. Many students experiment with alcohol during their University years, often as a social activity (2, 3). Its implications among University students are far-reaching. It encompasses academic consequences, including impaired cognitive function and decreased performance, as well as various health risks such as addiction, mental health issues, and physical harm (3–5). Moreover, alcohol-related behaviors can impact personal relationships, contribute to risky sexual practices, and pose a burden on University resources and the broader community (6–9).

According to recent global alcohol consumption statistics, 18.4% of the adult population aged 15 years and older engaged in alcohol consumption (10). Globally, alcohol consumption was accountable for 1.78 million deaths in 2020, founding it as the leading risk factor for mortality among males aged 15 to 49 years (6). The Global Burden of Disease (GBD) report in 2020 also estimated that approximately 1.34 billion individuals consumed harmful amounts of alcohol (6). The World Health Organization (WHO) has identified more than 300 diseases and adverse conditions that can result from irresponsible alcohol consumption patterns (1). The consumption of alcohol at any level is linked to health impairments caused by various diseases, such as liver cirrhosis, breast cancer, tuberculosis, and injuries (11–13). According to Global status report on alcohol and health, the WHO Collaborating Centre for Addiction and Mental Health made an estimation that in 2015, the adult population (aged ≥ 15 years) consumed approximately 6.43 liters of pure alcohol per capita (1).

In the Sub-Saharan African region, alcohol consumption among adolescents is also prevalent (4). The pooled prevalence of alcohol consumption among the Sub-Saharan Africans could be as high as 32.77%, while substance abuse among adolescents being 41.6% (4). However, the WHO Collaborating Centre for Addiction and Mental Health reported that, Sub-Saharan Africa presented distinctive findings, with the highest prevalence of heavy consumption (78.9%), even though the per capita consumption was relatively low at 4.14 liters (1).

Currently, there is a lack of adequate focus on alcohol consumption and its associated health consequences in developing countries. This problem has a particularly pronounced impact on the younger and more economically active segments of the population, who play a pivotal role in propelling a nation’s progress. Several observational studies have been conducted among students in developing country like Ethiopia, revealing varying prevalence rates of substance use, particularly alcohol consumption. For instance, in Ghana, the lifetime prevalence of alcohol consumption among University students was 39.5% (14), and in Nigeria the level of ever and current use was 43.5 and 31.1%, respectively, (9). Similarly, the prevalence of substance use among University students in Ethiopia varied from 16.7 to 72.6% (15, 16). Among college and University students, the lifetime prevalence of alcohol consumption ranges from 13 to 28% (16, 17).

Despite the growing concern surrounding alcohol consumption among University students in Ethiopia, there remains a noticeable research gap. Many existing studies tend to focus on students across all educational levels, including combinations of high school, college, and University students, and determining substance use behaviors, such as *Kchat* chewing, cigarette smoking, and alcohol consumption (2, 16, 18, 19). This approach lacks a comprehensive national perspective, particularly with regard to University students. Notably, there is no research available that assesses the extent of alcohol consumption among university students in Ethiopia on a national level. Therefore, this systematic review and meta-analysis provide critical insights into alcohol consumption patterns and associated factors among university students in Ethiopia. Addressing these research gaps is crucial for developing evidence-based strategies that account for the complexity of alcohol consumption among Ethiopian university students and ultimately improving their well-being.

## Methodology

### Study registration

The systematic review and meta-analysis followed the Joanna Briggs Institute (JBI) methodology for systematic reviews and Meta-analysis (20). The protocol has been registered in PROSPERO (CRD42023444392).

### Search strategy

All potentially relevant articles, gray literature, and government reports were meticulously searched to conduct this study. International databases and libraries, such as PubMed/MEDLINE, Google Scholar, Hinari, African Journals Online and Science direct, were included to search relevant studies. We retrieved gray literature using Google and Google Scholar searches. We also reviewed reference lists of identified studies in order to find and retrieve additional articles. Unpublished studies were retrieved from the official websites of international and local organizations and universities. An extensive search was done from the databases using the following keywords: “prevalence,” “proportion,” “magnitude,” “alcohol,” “alcohol consumption,” “alcohol drinking,” “alcohol user,” “substance user,” “factors,” “determinants,” “predictors,” “factors associated,” “associated factors,” “risk factor,” “university student,” “students,” and “Ethiopia” without restring the study period. We undertook advanced search by combining the search terms using Boolean operators (Supplementary 1). The search for all articles was conducted from September 1st to 15th October, 2024.

### Study selection and eligibility criteria

The review included Ethiopian studies that reported the prevalence of alcohol consumption, as well as articles published in scientific journals and gray literature. Only study reports written in English language and full-text articles were considered. Furthermore, the review looked at all observational study (cross-sectional). Articles that could not be accessed for full text after at least three emails to the primary study’s principal investigators were excluded. Two independent reviewers (SAD and CD) screened the records for inclusion. Each reviewer carried out the screening evaluation independently. Disagreements among reviewers were discussed with the review team members until a consensus is reached.

### Inclusion criteria

Population: This systematic review and meta-analysis included studies conducted among University students in Ethiopia.

Exposure: An individual student who experienced alcohol consumption.

Comparison: Individual students who drunk and do not drink alcohol at University.

Outcome: This study estimated the pooled magnitude of alcohol consumption and its association among University students. The magnitude was computed as the proportion of the number of individuals who consume alcohol to the total number of participants and multiplied by 100.

Study setting: Ethiopia.

Study design: observational study (cross-sectional, cohort and case–control).

Publication: Both published and unpublished studies were included.

### Exclusion criteria

Qualitative studies, unretrievable studies, editorial letters, studies with poor methodological quality, and studies that did not report the outcome of interest were excluded from the meta-analysis.

### Outcome assessment

The primary outcome of this meta-analysis was to estimate the overall prevalence of alcohol consumption among university students in Ethiopia. This was calculated by dividing the number of alcohol users by the total sample size and multiplying by 100. Additionally, the study aimed to identify factors associated with alcohol consumption, expressed as a log odds ratio.

### Quality assessment and data extraction

**Figure 1:**
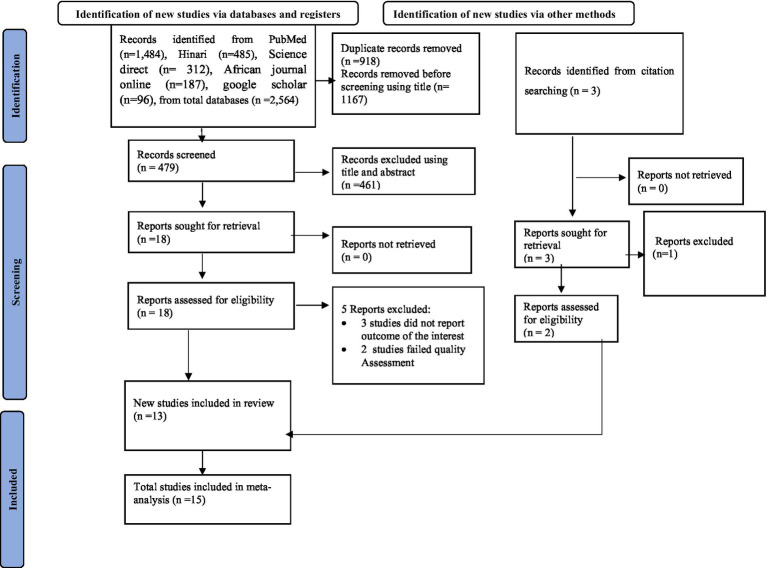
PRISMA flow diagram of the included studies for the systematic review and meta-analysis of alcohol consumption and its associated factors among university students in Ethiopia, 2024.

Duplicated records were removed using reference management software (EndNote X7). The titles and abstracts were reviewed by two reviewers (SAD and CD) and records that did not fulfill our criteria, such as guidelines, reviews, position papers, and records without an abstract (if title is the only available information), were excluded. The Joanna Briggs Institute Meta-Analysis of Statistics Assessment and Review Instrument (JBI-MAStARI) was used to assess papers objectively (21). When there is disagreement between reviewers, it was discussed with the other members of the review team until an agreement is reached. A third reviewer was invited to resolve the disagreements between the two independent reviewers (MG). A standardized data extraction format was used by three independent reviewers to extract the data (SAD, CD and MG). The data extraction spread sheet included primary author name, year of publication, study setting (region), study design, sample size, response rate, alcohol consumption (Yes/No), and quality of the paper result. PRISMA flow diagram was used to show the review process of the studies (Figure 1).

### Assessing certainty in the findings

The Grading of Recommendations, Assessment, Development, and Evaluation (GRADE) approach for assessing the quality of evidence was used to create the summary of findings. The outcomes reported in the summary of findings were the pooled prevalence of alcohol consumption among university students and its association with different factors. The quality of the evidence was assessed independently based on the five domains established by the GRADE guidelines: risk of bias, inconsistency, indirectness, imprecision, and publication bias.

### Data analysis and synthesis

Using the ‘generate’ command in STATA 14, from the original research, odds ratios (OR) and standard errors were created for all the included studies. The *p*-values of the Cochrane Q-test and I^2^-test statistics were used to assess the heterogeneity among studies. The publication bias was assessed visually using a funnel plot. Asymmetry in the funnel plot indicates the possibility of publication bias. Moreover, Egger’s test was used to determine if there has been a significant publication bias, with a p-value of less than 0.10 indicating the presence of significant publication bias (22). Because Egger’s test is more specific than Begg’s test, it was used to assess publication bias. We used the log odds ratio to determine the association between different factors and their alcohol consumption. Furthermore, sensitivity analysis using a random effects model was performed to assess the effect of a single study on the pooled magnitude estimates (23). Sub-group analysis was used to lower the random variations between the primary study’s inter-group estimates, and the analysis was done based on region, year of publication and sampling technique.

## Results

### Identification and selection of studies

Upon completing the electronic database search, 2,564 articles were identified. Using the Endnote reference manager, 461 duplicate studies were excluded using title and abstract. Moreover, 5 studies were excluded based on the quality assessment and outcomes of the interest. Finally, 15 studies were eligible for this meta-systematic review and analysis. The process of selecting these studies is visually described in a PRISMA flow diagram (Figure 1).

### Characteristics of the included studies

We included 15 studies in meta-analysis to assess the prevalence of alcohol consumption among university students in Ethiopia. These studies involved a total of 10,500 individuals, with sample sizes ranging from 188 to 1,239 participants across the various studies. Notably, the study findings indicated that the highest lifetime prevalence of alcohol consumption was reported in Dire-Dawa University (24), at 60%. Conversely, the lowest lifetime prevalence, at 25.1%, was documented in a study conducted at Mekelle University (Table 1) (25). The findings concerning alcohol consumption varied among the included studies and did not provide conclusive results.

**Table 1:** Characteristics of studies included in the systematic review and meta-analysis of alcohol consumption and associated factors among university students in Ethiopia.

Authors	Year of publication	Region	Study design	Sampling technique	Sample size	Response rate	Lifetime prevalence	Current prevalence	Quality scores (8)	Reference
Chekole	2020	SNNPR	Cross-sectional	SytRS	876	91.70%	41.8	24.4	7	(31)
Deressa and Azazh	2011	AA	Cross-sectional	Not available	632	98.40%	31.4	4.5	6	(32)
Gebreslassie et al.	2013	Tigray	Cross-sectional	MSST	764	98.70%	34.5	9.3	6	(7)
Birhanu	2016	Oromia	Cross-sectional	SRS	440	97%	50.7	26.1	8	(8)
Esmael	2014	Oromia	Cross-sectional	MSST	845	96.6%	35	-	7	(33)
Gebremariam et al.	2018	Amhara	Cross-sectional	SS	695	89.00%	36.3	17	7	(15)
Aklog et al.	2013	Amhara	Cross-sectional	SRS	423	97.00%	-	13.4	6	(34)
Tesso Kumburi et al.	2017	DD	Cross-sectional	SytRS	1,239	73.85%	60	45	8	(24)
Tesfaye et al.	2014	Oromia	Cross-sectional	MSST	1,040	98.30%	50.2	20	7	(35)
Kassa et al.	2016	Sidama	Cross-sectional	MSST	590	94.50%	48.7	29.5	7	(36)
Shiferaw	2017	Somale	Cross-sectional	MSST	648	92.60%	27.3	18.3	6	(37)
Derese et al.	2014	Oromia	Cross-sectional	SRS	764	94.90%	41.7	17.5	6	(38)
Desta et al.	2018	Oromia	Cross-sectional	Census	188	98.90%	26.34	25.3	8	(39)
Kumesa et al.	2015	Ethiopia	Cross-sectional	SRS	355	97.70%	40.2	35.6	6	(40)
Hagos et al.	2016	Tigray	Cross-sectional	SRS	271	100.00%	25.1	12	7	(25)
Adere et al.	2017	Amhara	Cross-sectional	MSST	730	89.70%	33.1	27.9	7	(41)

MSST, multi-stage sampling technique; SS, stratified sampling; SytRS, systematic random sampling technique; SRS, simple random sampling technique; DD, Dire Dawa; AA, Addis Ababa.

### Prevalence of alcohol consumption

**Figure 2:**
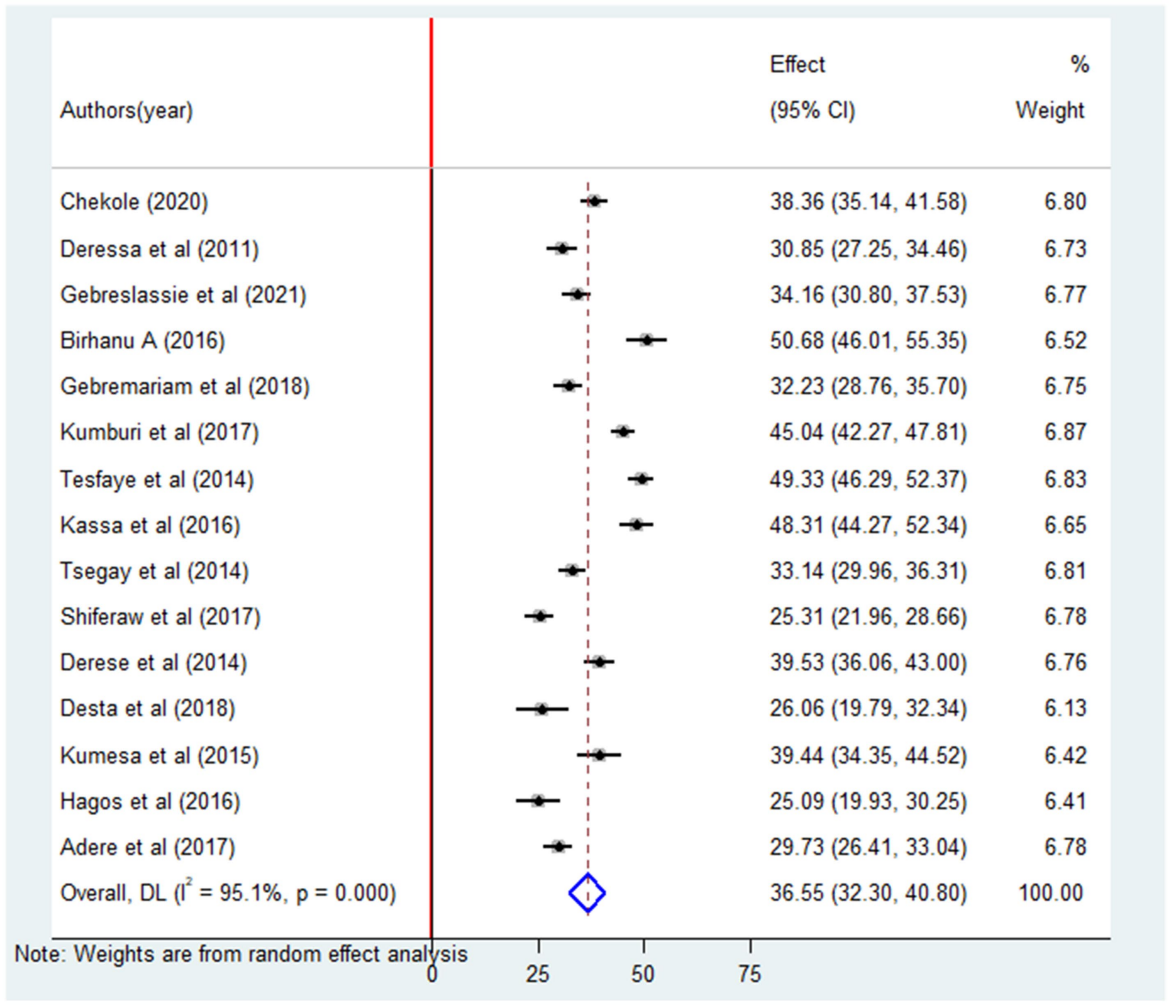
Forest plot of the pooled prevalence of lifetime alcohol consumption among university students in Ethiopia, 2024.

**Figure 3:**
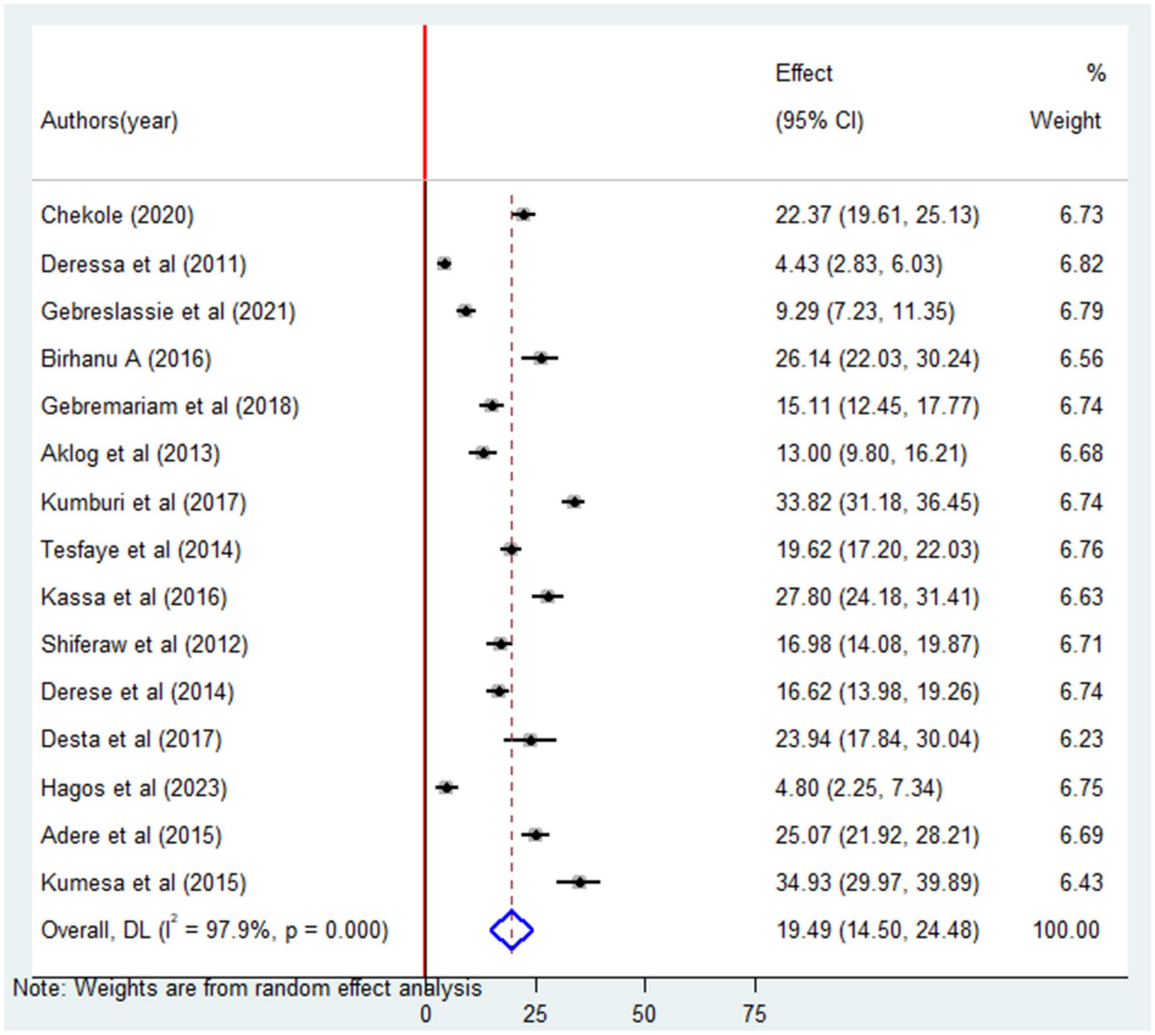
Forest plot of the pooled prevalence of current alcohol consumption among university students in Ethiopia, 2024.

The analysis of 15 studies revealed that the pooled prevalence of lifetime alcohol consumption among university students was 36.55% (95% CI: 32.30–40.80). A significant degree of heterogeneity (*I*^2^ = 95%; *p* = 0.00) was observed among these studies (Figure 2). Thus, we applied a random effects model to estimate the overall prevalence of alcohol consumption among university students. Furthermore, the pooled prevalence of current alcohol consumption among university students was 19.49% (95% CI: 14.50–24.48) (Figure 3).

### Publication bias

**Figure 4:**
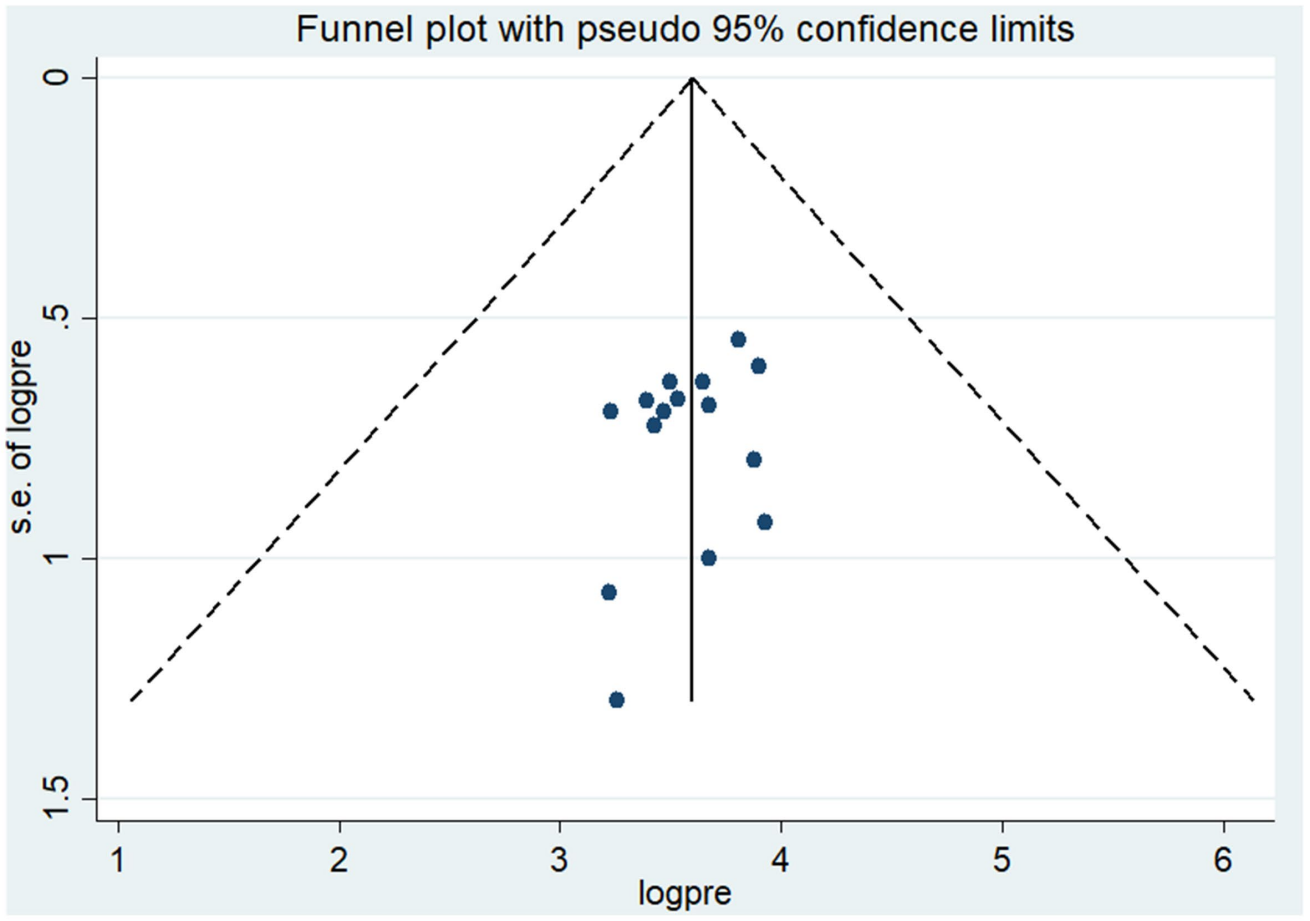
Funnel plot of the pooled prevalence of lifetime alcohol consumption among university students in Ethiopia, 2024.

To assess publication bias, we employed both funnel plots and statistical tests, including the Egger and Begg’s tests, with a significance level set at 5%. Upon visual check, the funnel plot showed symmetrical distribution, suggesting no apparent signs of publication bias. Additionally, the Egger test statistics did not indicate the presence of statistically significant publication bias (*p* = 0. 307). Furthermore, there is no substantial presence of small-study effects based on the test results (Figure 4).

### Sensitivity analysis

**Figure 5:**
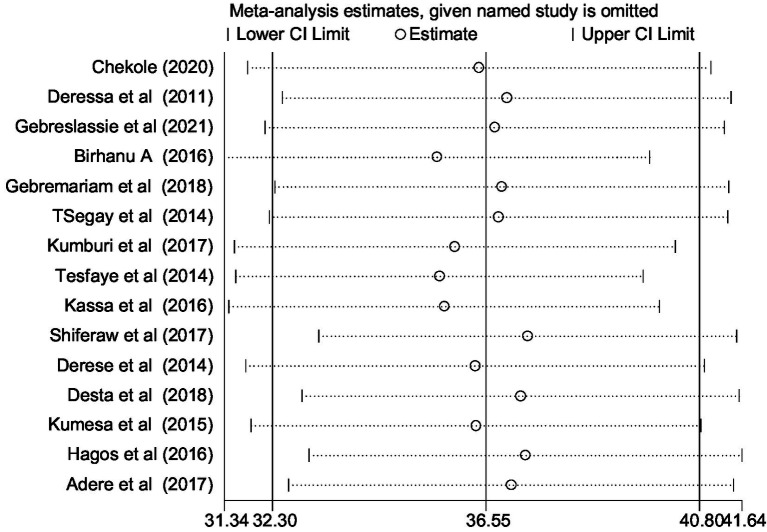
Sensitivity analysis for single study influence on overall pooled estimated of tobacco smoking among university students in Ethiopia, 2024.

To evaluate the potential impact of individual studies on the overall meta-analysis estimate, we performed a sensitivity analysis using a random effects model. This analysis was conducted to discern whether any specific study significantly influenced the overall pooled estimate of alcohol consumption. The outcomes of this sensitivity analysis revealed that none of the individual studies had a substantial effect on the overall pooled prevalence (Figure 5).

### Subgroup analysis by year of publication

**Figure 6:**
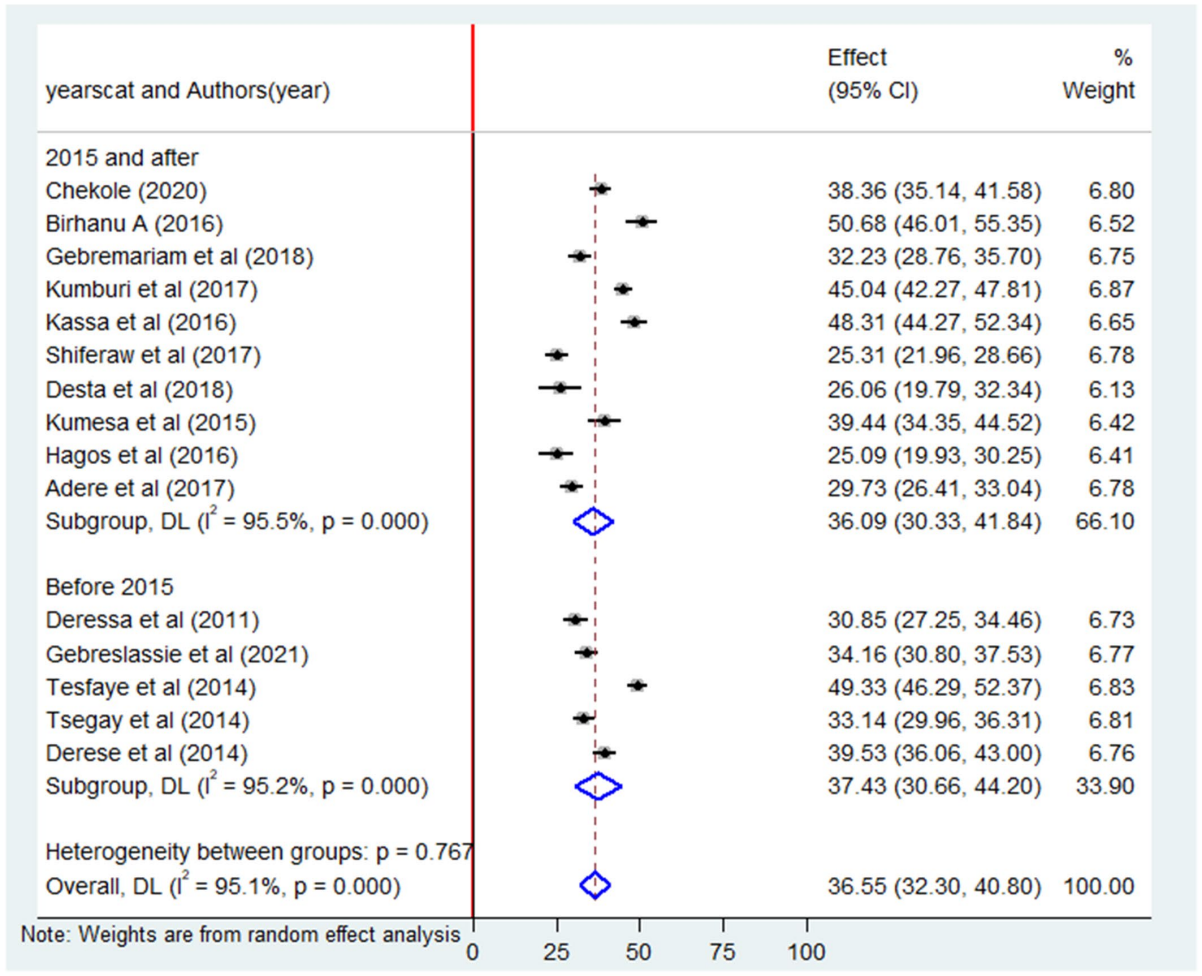
Subgroup analysis by year publication of the pooled prevalence of lifetime alcohol consumption among university students in Ethiopia, 2024.

The objective of this analysis was to compare the pooled prevalence of lifetime alcohol consumption among university students in Ethiopia between studies published prior to and after 2015. The findings of the subgroup analysis demonstrated that the pooled prevalence of lifetime alcohol consumption in studies conducted before 2015 was 37.43% (95% CI: 30.66–44.120), slightly exceeding the prevalence observed in studies conducted during and after 2015 was 36.09% (95% CI: 30.33–41.84) (Figure 6).

### Subgroup analysis by region

**Figure 7:**
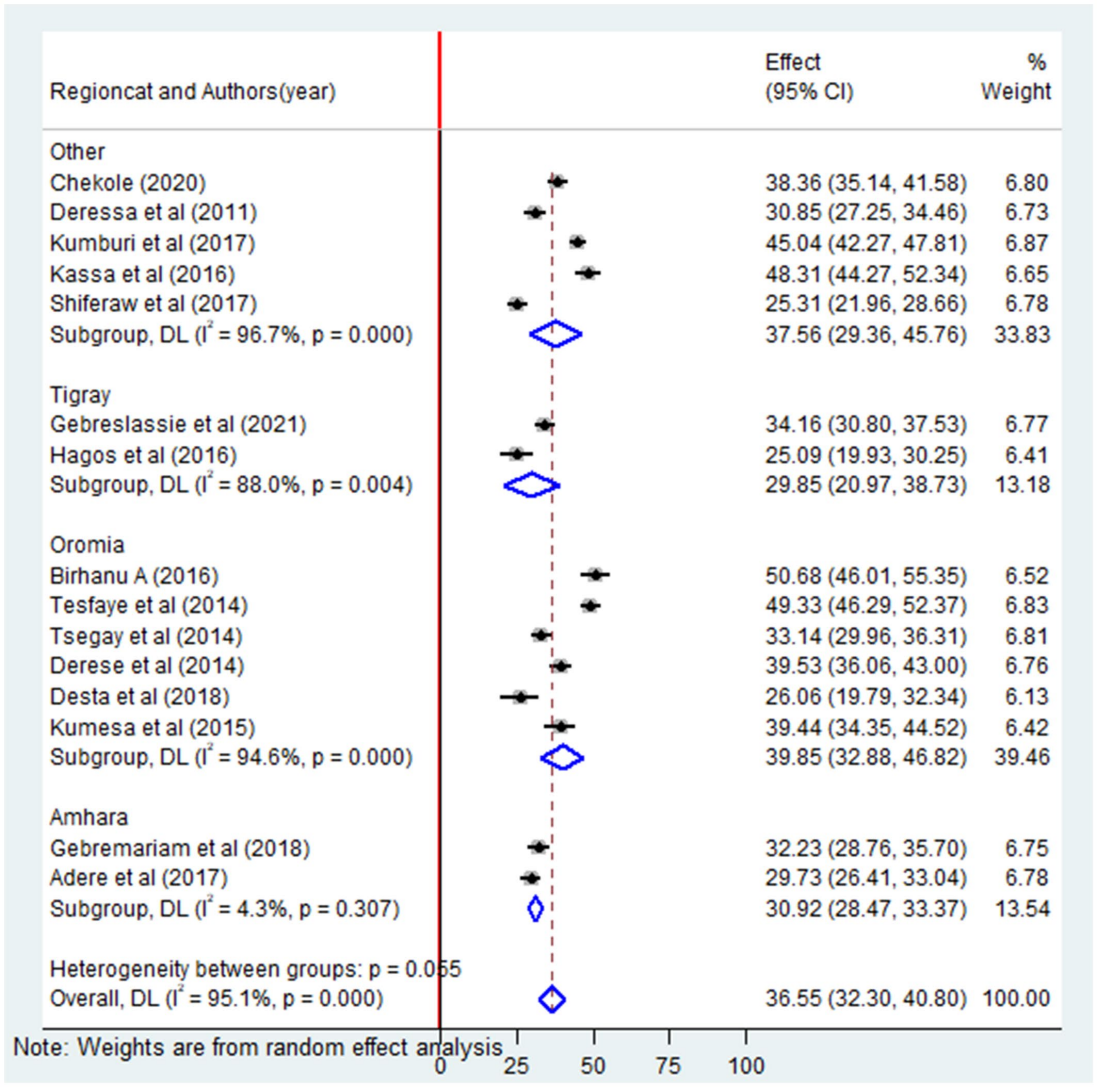
Subgroup analysis by study countries of the pooled prevalence of lifetime alcohol consumption among university students in Ethiopia, 2024.

Furthermore, a subgroup analysis was carried out based on the region to reduce the possibility of random variations across studies and to assess the prevalence of lifetime alcohol consumption in various regions. The highest pooled prevalence of alcohol consumption was observed in the Oromia region at 39.8% (95% CI: 32.8–46.8), were as the lowest were observed in Tigray at 29.8% (95% CI: 20.9–38.7) (Figure 7). The results of this analysis indicated that there were significant differences in the pooled prevalence of alcohol consumption among university students across different region. These findings emphasize that there is significant variation in alcohol consumption among university students from different region of Ethiopia (Figure 7).

### Subgroup analysis by study technique

**Figure 8:**
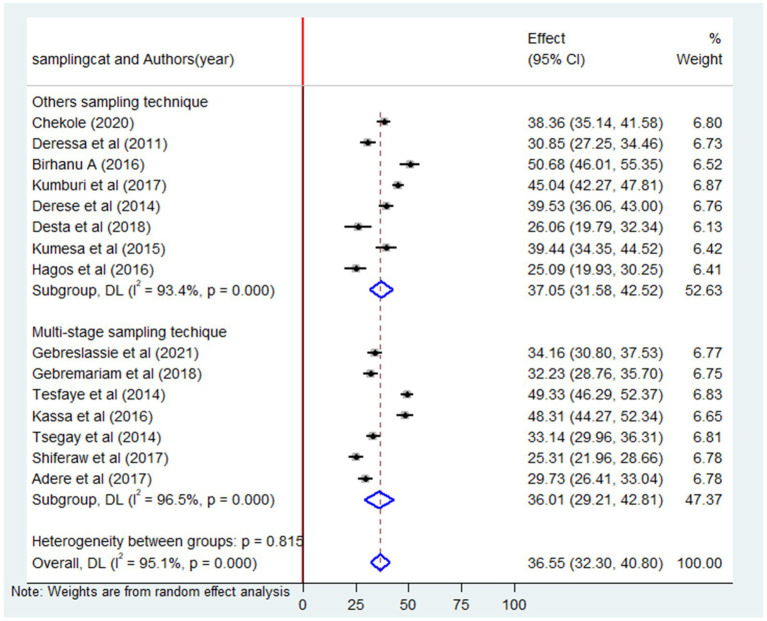
Subgroup analysis by sampling technique of the pooled prevalence of lifetime alcohol consumption among university students in Ethiopia, 2024.

The findings from the subgroup analysis unveiled that the combined prevalence of lifetime alcohol consumption among university students in Ethiopia was 36% (95% CI: 29.21–42.81) for studies employing a multi-stage study technique. This figure was slightly lower than the pooled prevalence observed in studies using other sampling technique (Table 1) which stood at 37% (95% CI: 31.5–42.5) as depicted in Figure 8.

### Factors associated with alcohol consumption

Among the 15 studies that fulfilled the inclusion criteria, five studies were considered for assessing the association between alcohol consumption and having a friend currently drinking alcohol. Students who had friends currently drinking alcohol were 1.75 times likely to engage in alcohol consumption than those who did not have such friends (OR = 1.75, CI: 95%, 1.29–2.21). Students in universities who smoke cigarette were also twice as likely to consume alcohol compared to non-smokers in four studies (OR = 1.92, CI: 95%, 1.06–2.78). Six studies were considered for assessing the association between alcohol consumption, and having a family currently drinking alcohol and chewing *Kchat*. Additionally, students who had family members who drank alcohol were 1.14 times more likely to consume alcohol compared to those from families without these behaviors (OR = 1.14, CI: 95% 1.1–1.7). Furthermore, the Chewing of *Kchat* was identified in six studies as additional factors influencing alcohol consumption. Students in universities who consume *Kchat* were 1.7 times likely to consume alcohol compared to non-consumer (OR = 1.7, CI: 95%, 1.23–2.16) (Table 2).

**Table 2:** The pooled effect size of factors associated with alcohol consumption among university students in Ethiopia, 2024.

Variables	Number of study included	Odd ratio (95% CI)	Heterogeneity	
			*I* ^2^	*p*-value
Male	9	1.17 (0.96–1.38)	66.8%	0.002
Friend currently drinking alcohol	5	1.75 (1.29–2.21)*	71.1%	0.008
Cigarette smoking	4	1.92 (1.06–2.78)*	37.1%	0.189
Urban residence	2	1.29 (0.82–1.76)	0.0%	0.543
Peer pressure	2	1.36 (0.73–1.98)	0.0%	0.33
Illegal drug use	2	1.59 (−1.60–4.80)	10.4%	0.291
Family’s drinks alcohol	6	1.14 (1.10–1.72)*	66.4%	0.011
*Kchat* use	6	1.70 (1.23–2.16)*	43.5%	0.115

^*^Significant at *p*-value <0.05.

## Discussion

Numerous studies have explored the potential link between alcohol use and its associations with various factors. However, the results of these studies have often been conflicting and lacking in consensus. The findings from our analysis of 15 studies encompassing 10,500 university students in Ethiopia shed light on the prevalence and various factors associated with alcohol consumption. Notably, the pooled prevalence of lifetime alcohol consumption was 36.5%, underlining the significance of this issue among university students in the country. The substantial impact of alcohol consumption identified in the current systematic review and analysis exceeds the figures reported in global alcohol consumption statistics (18.4%) and the global status report on alcohol and health in Sub-Saharan Africa (32.7%), systematic review and meta-analysis on alcohol consumption among all students (26.19%), and a report from photoactive substance use among University Students (32.28%) (1, 2, 10, 16). Our findings show the urgent need for targeted interventions and preventive measures to address the high prevalence of alcohol consumption among university students in Ethiopia. This is a pressing public health concern that demands focused attention and strategies to the specific context of university life in the country. Nonetheless, the outcome of this systematic review reveals a lower prevalence of alcohol consumption compared to other sources of evidence in Ethiopia. For instance, epidemiological data on alcohol consumption in Ethiopia indicates a rate of 44.16% (26). Likewise, a report from prevalence of life time substance use specifically alcohol consumption among students in Ethiopia was reported to be 46.2% (19). The lower prevalence rate in the current systematic review compared to other studies may be due to a combination of factors related to sampling, timing, and participant characteristics.

This comprehensive analysis unveiled a significant association between alcohol consumption and several factors, including peer networks, tobacco use, *Kchat* chewing, and family influences. Peer influence emerged as a potent factor, as students with friends currently consuming alcohol were 1.75 as likely to engage in alcohol consumption. This outcome is consistent with the findings of a systematic review and meta-analysis on psychoactive substance abuse and alcohol consumption among both high school and University students, and adolescent and Youth in Ethiopia (16, 27). Peer influence significantly impacts alcohol consumption, driven by factors such as social norms, direct peer pressure, modeling behavior, social events, and availability of alcohol. Peer networks and social dynamics play pivotal roles in shaping students’ drinking habits, necessitating peer-led initiatives and awareness programs (26). This influence can be reinforced by peer identity, positive reinforcement, and social support. The desire to conform and meet peer expectations can lead individuals to engage in drinking behaviors within their social circles. So, it is essential for designing interventions that promote responsible drinking and mitigate negative consequences associated with excessive alcohol use among peers.

The link between tobacco use and alcohol consumption was another significant finding, with students who smoked cigarettes having twice the likelihood of alcohol consumption. This finding is consistent with the findings of a systematic review and meta-analysis for substance use among adolescents in sub-Saharan Africa (4). This finding is also aligns with systematic review and meta-analysis conducted in Ethiopia that have documented significant associations between smoking cigarette and alcohol consumption (27–29). Smoking cigarettes can significantly influence the alcohol consumption of University students (29). The co-occurrence of these behaviors is common due to shared addictive qualities. Nicotine in cigarettes can enhance the pleasurable effects of alcohol, leading to increased alcohol consumption (29). Additionally, students who smoke may find themselves in social contexts where both smoking and drinking are prevalent, further reinforcing this association (28, 29). It is important for addressing substance use and promoting healthier behaviors among university students. This suggests that substance use behaviors often co-occur and underscores the importance of addressing multiple substances in prevention efforts.

Family environment was identified as the determinant factors of alcohol consumption. Students from families with a history of alcohol consumption are 1.14 times more likely to consume alcohol compared to their counterparts. Family members who drink alcohol can significantly impact students’ alcohol consumption (19, 26). Younger age children, such as students might be tempted with the presence of alcohol at household level. Exposure to these behaviors in the family environment may normalize and increase the likelihood of alcohol use. Additionally, family dynamics and communication patterns play a role in shaping attitudes and behaviors toward alcohol use. Recognizing these familial influences is vital for targeted interventions addressing alcohol consumption among students. Family-based interventions and support for creating alcohol-free home environments could prove beneficial.

Moreover, the Chewing *Kchat* was identified as additional factor affecting alcohol consumption. This finding is also aligns with systematic review and meta-analysis conducted in Ethiopia that have documented significant associations between chewing *Kchat* and alcohol consumption (27). Furthermore, the results of the our systematic review align with the findings from a systematic review and meta-analysis on *Kchat* chewing among University students in Ethiopia, indicating that chewing *Kchat* can significantly influence students’ alcohol consumption (28). Social settings where chewing *Kchat* occurs can normalize alcohol consumption (30). Students may also use alcohol to counteract the effects of *Kchat*. These findings highlight the complexity of substance use behaviors among university students, necessitating a comprehensive approach to address multiple risk factors simultaneously.

### Strengths and limitations of the study

One of the strengths of the current meta-analysis is that it follows the updated Preferred Reporting Items for Systematic Reviews and Meta-Analyses (PRISMA) guidelines. However, a limitation of this meta-analysis is that it did not establish causality between the independent and dependent variables, as most of the included studies utilized cross-sectional designs.

## Conclusion

This systematic review and meta-analysis provided insights into alcohol consumption among university students in Ethiopia. The pooled prevalence of alcohol consumption was found to be 36.5%. The study underscores the multifaceted nature of alcohol consumption among university students; peer influence, smoking habits, family environment, and other substance use behaviors all playing significant roles in shaping alcohol consumption patterns. These results emphasize the importance of considering peer networks, tobacco use, and family influences when addressing alcohol consumption among university students. Additionally, the study highlights the need for comprehensive interventions that take into account these multifaceted factors to promote responsible alcohol use among this population. Future research and targeted programs should address these complex factors to develop effective strategies for reducing risky alcohol behaviors among university students.

## Data Availability

The original contributions presented in the study are included in the article/Supplementary material, further inquiries can be directed to the corresponding authors.
