# Supplementary material for: Alcohol consumption among university students in Ethiopia: a systematic review and meta-analysis of epidemiological studies
Source: Front Public Health. 2025 Sep 9;13:1513242. doi: 10.3389/fpubh.2025.1513242 (PMC12454427; doi:10.3389/fpubh.2025.1513242)
Supplement: Supplementary file 1 [file Table_1.docx]

| Mesh heading | Combination | Number of article | Last search date | Electronic data base |
| --- | --- | --- | --- | --- |
|  | ((((alcohol consumption[Tiab]) OR (alcohol use[Tiab])) OR (substance abuse [Tiab])) AND (university students [Tiab])) AND (ethiopia [Tiab]) | 30  1454 | 15/10/2024 | PubMed |
|  | (((((((((((prevalence of alcohol consumption ) OR (alcohol user)) OR (alcohol drinking)) OR (substance abuse)) OR (substance abuse user)) AND (associated factors)) OR (risk factors)) OR (predictors)) OR (factors associated)) AND (university students)) OR (students)) AND (Ethiopia) |  |  |  |
|  | ((prevalence of alcohol consumption) OR (alcohol user) OR (alcohol drinking) OR (substance abuse) OR (substance abuse user)) AND ((associated factors) OR (risk factors) OR (predictors) OR (factors associated)) AND ((university students) OR (students)) AND (Ethiopia) | 485 | 15/10/2024 | Hinari |
|  | Alcohol consumption and associated factors among university students in Ethiopia | 312 | 15/10/2024 | Science Direct |
|  | Alcohol consumption and associated factors among university students in Ethiopia | 96 | 15/10/2024 | Google scholar |
|  | Alcohol consumption and associated factors among university students in Ethiopia | 187 | 14/10/2024 | African journal online |
